# How Is Professional Identity Associated with Teacher Career Satisfaction? A Cross-Sectional Design to Test the Multiple Mediating Roles of Psychological Empowerment and Work Engagement

**DOI:** 10.3390/ijerph19159009

**Published:** 2022-07-25

**Authors:** Binghai Sun, Feng Zhu, Shuwei Lin, Jiayu Sun, Ying Wu, Weilong Xiao

**Affiliations:** 1 Research Center of Tin Ka Ping Moral Education, Zhejiang Normal University, Jinhua 321004, China; jky18@zjnu.cn (B.S.); zjsfzf@zjnu.edu.cn (F.Z.); lin_shuwei@zjnu.edu.cn (S.L.); wy8305@zjnu.edu.cn (Y.W.); 2 College of Teacher Education, Zhejiang Normal University, Jinhua 321004, China; 3 College of Education and Human Development, Zhejiang Normal University, Jinhua 321004, China; 4 Key Laboratory of Intelligent Education Technology and Application of Zhejiang Province, Zhejiang Normal University, Jinhua 321004, China; 5 American Heritage School, American Fork, UT 33484, USA; rain.sun@berkeley.edu

**Keywords:** career satisfaction, professional identity, psychological empowerment, work engagement, teachers

## Abstract

(1) Purpose: Previous studies investigated the positive relationship between professional identity and career satisfaction in teachers, but the underlying reasons were not explored. Therefore, the present study explores the mediating effects of two variables, namely, psychological empowerment and work engagement on the relationship between professional identity and career satisfaction. (2) Method: The present study used the professional identity scale, psychological empowerment scale, Utrecht Work Engagement scale and career satisfaction scale to investigate 2104 teachers (Mage = 39.50 years, SD = 8.74) in a province in China. The demographic variables (e.g., gender, age, teaching age) were controlled as covariates to conduct conservative predictions. (3) Result: (a) professional identity is positively related to career satisfaction; (b) psychological empowerment and career satisfaction play parallel mediator roles between professional identity and career satisfaction; (c) psychological empowerment and career satisfaction play serial mediator roles between professional identity and career satisfaction. (4) Limitations: Data were collected by participant self-report. This method may lead to recall bias. Further, we adopted a cross-sectional rather than experimental or longitudinal design, thus precluding causal conclusions. Lastly, it would be useful to validate our findings with a national sample. (5) Conclusions: The present study indicates that the relationship between professional identity is positively associated with teacher career satisfaction. More importantly, professional identity can indirectly make an impact on teacher career satisfaction through the single mediating effects of psychological empowerment and work engagement, and the chain mediating effect, by improving the level of psychological empowerment, and thereby increasing work engagement.

## 1. Introduction

“The root of goodness is teaching, and the root of teaching is teachers.” Teachers are the foundation and source of education. Teachers’ career satisfaction refers to teachers’ intrinsic evaluation and definition of their career outcomes, which reflect their overall satisfaction with their career experience [1,2]. Teacher career satisfaction is an essential factor affecting teacher professional development.

Previous studies showed that teachers career satisfaction can be associated with teacher turnover intentions; teachers with lower career satisfaction were more likely to resign; and improving teacher career satisfaction could effectively improve work enthusiasm and reduce their turnover intention [3,4,5,6]. Moreover, teachers career satisfaction is related to their well-being and work enthusiasm, and closely related to the quality of education [6,7]. Based on the importance of teacher career satisfaction, it is indispensable to explore the factors that can improve teachers career satisfaction to achieve the purpose of improving teaching quality.

### 1.1. Professional Identity and Career Satisfaction

Teacher professional identity refers to a teacher’s optimistic attitude and a strong sense of commitment to the profession, reflected in the teacher’s desire to maintain their profession and the degree of liking. Avalos and Aylwin found that teachers professional identity could reflect their professional lives and career decision-making [8]. Some empirical research showed that professional identity was associated with career satisfaction [9,10,11]. For example, Sabanciogullari and Dogan conducted research in Turkey to explore factors influencing the turnover intention of nurses; their research found that nurse professional identity could affect their job satisfaction [10].

According to Social Identity Theory (SIT), an individual’s desire is to establish and maintain a positive self-image. SIT indicates that an individual’s identification with their occupation could promote career satisfaction [12]. Based on the theory and empirical research, we confirmed that professional identity is related to career satisfaction, but the underlying reasons for this were not explored in depth. Therefore, the present study focuses on exploring why professional identity affects teachers career satisfaction. Based on previous studies [9,10,12], in the present study we hypothesize that teacher professional identity is associated with their career satisfaction (H1).

### 1.2. Professional Identity, Psychological Empowerment, and Career Satisfaction

Psychological empowerment may play a mediating role between professional identity and career satisfaction. Psychological empowerment refers to the synthesis of psychological states or cognition experienced by an individual [12,13,14,15]. Previous research found that both professional identity [12,16,17] and career satisfaction [18,19,20] are positively correlated with psychological empowerment. According to Cognitive Evaluation Model Theory (CEMT), individuals evaluate themselves and their career condition in conjunction with their own and others’ perspectives, and individuals with positive psychological empowerment would gain a stronger sense of meaning in their work [12,13]. We, therefore, believe that professional identity is related with psychological empowerment, and several empirical studies confirmed this view [17,21].

Meanwhile, other studies also found that individuals with higher levels of psychological empowerment tend to exhibit higher career satisfaction [18,22,23,24]. For example, Blais et al. analyzed the dimension of work fatigue among military and civilian employees. They found professional identity was positively correlated with the career satisfaction of military employees. These results indicate that psychological empowerment may play a mediating role between professional identity and career satisfaction. Hence, the present study suggests that the relationship between professional identity and career satisfaction may be mediated by psychological empowerment (H2).

### 1.3. Professional Identity, Work Engagement, and Career Satisfaction

Work engagement may play a mediating role between professional identity and career satisfaction. Work engagement refers to an individual’s cognitive preoccupation, engagement, and concern with their current work, reflecting their identification with their present work [15,25,26]. According to Cognitive Dissonance Theory (CDT), when an individual holds an attitude of professional identity, they tend to show behaviors that are consistent with this attitude so as to achieve a consistency of attitudes and behaviors, thereby reducing the tension caused by inconsistent attitudes and behaviors. Consequently, individuals with high levels of professional identity are bound to exhibit more positive work attitudes and behaviors [27,28]. Indeed, several empirical studies confirmed this opinion, that is, individuals with high professional identity show higher work engagement [29,30,31].

Meanwhile, research found work engagement to be significantly associated with career satisfaction [32,33,34,35], especially the dedication component of work engagement [32]. For example, Fute et al. explored teacher satisfaction during the COVID-19 pandemic and found that both teacher work values and work engagement during the pandemic could significantly predict teacher job satisfaction; they also found that work engagement played a mediating role between a teacher’s work values and job satisfaction [35]. Additionally, and basing their research on Social Identity Theory (SIT), Karanika-Murray et al. examined the correlation between organizational identity, work engagement, and job satisfaction. They found that all three factors of work engagement (i.e., vigor, dedication, focus) were positively related to job satisfaction. Previous research, therefore, suggests that work engagement may be an important mediator, affecting the correlation between professional identity and career satisfaction. Accordingly, this study hypothesizes that work engagement plays a mediating role between professional identity and career satisfaction (H3).

### 1.4. Professional Identity, Psychological Empowerment, Work Engagement, and Career Satisfaction

Furthermore, we propose that psychological empowerment and work engagement may mediate the correlation between professional identity and career satisfaction. Under the serial mediation model, professional identity leads to higher psychological empowerment, more challenging work, and more engagement in work. Thus, the three variables (i.e., professional identity, psychological empowerment, and work engagement) are related to teacher career satisfaction. Previous studies indirectly support the serial mediation model, with research showing a positive correlation between psychological empowerment and work engagement [36,37,38]. For example, Meng and Sun explored whether psychological empowerment is positively associated with the work engagement of university staff. Their findings showed that psychological empowerment can significantly predict university staff work engagement and is mainly achieved through the two dimensions of capability and meaning [37]. Lu and Bai investigated the impact of job crafting of scientific research institution intellectual property personnel on career satisfaction. They found that work engagement was significantly associated with career satisfaction: it had a mediating role between job crafting and career satisfaction [39].

Meanwhile, other previous research indicated that professional identity is associated with career satisfaction [12,21]. These results indicated that psychological empowerment and work engagement might mediate the correlation between professional identity and career satisfaction. In our study, we, therefore, presume that psychological empowerment and work engagement have an impact on the role of professional identity on teacher career satisfaction in a serial mediation model (H4).

### 1.5. The Present Study

In the present study, we verify the hypothesis from two main aspects. On the one hand, the present study aimed to explore how teacher professional identity is associated with their career satisfaction and the underlying mechanisms. On the other hand, we planned to investigate whether psychological empowerment and career satisfaction play parallel and multiple mediating roles in the relationship between professional identity and teacher career satisfaction. Based on these theories and existing empirical evidence, we hypothesized:

(1) Professional identity is positively related to career satisfaction.
(2) Psychological empowerment and career satisfaction are parallel mediators between professional identity and career satisfaction.
(3) Psychological empowerment and career satisfaction are mediators between professional identity and career satisfaction.

**Figure 1:**
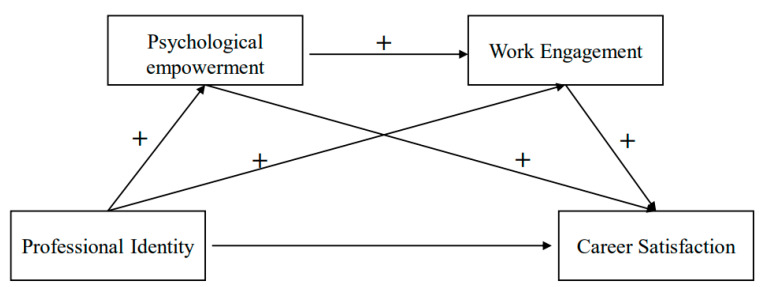
The multiple mediation model.

All hypotheses are shown in Figure 1. Many empirical studies demonstrated that professional identity is associated with career satisfaction [9,10]. However, the internal mechanisms between professional identity and career satisfaction are still unknown. In addition to integrating multiple mediator variables and understanding the information between them, the multiple mediation model can comprehensively examine the complex processes and mechanisms of how independent variables affect dependent variables. Since the multiple mediation model has advantages over the simple model, this study constructs a multiple mediation model to explore the underlying mechanism between variables.

## 2. Method

### 2.1. Participants

A total of 2014 kindergarten, primary school, middle school, and high school teachers in Zhejiang province, China were identified as the final sample (valid response rate: 89.91%). We randomly selected teachers at different stages of different types of schools (public schools and private schools) in developed and underdeveloped areas of Zhejiang Province as participants in this experiment. The final sample consisted of 624 male teachers (29.70%) and 1480 female teachers (70.30%), with an average age of 39.50 years (*SD* = 8.76) and the average teaching experience of 17.99 years (*SD* = 9.64). A total of 1746 (83.00%) teachers had majored in a teaching program, 312 (14.80%) teachers had majored in a non-teaching program, and information about their major was missing for 46 (2.20%) teachers. By “teaching program”, we mean a specialist teacher-training program (i.e., for primary, middle, or secondary teachers).

### 2.2. Measurement Tools in the Current Study

#### 2.2.1. Career Satisfaction

Teacher career satisfaction was assessed using a five item, 5-point Likert scoring system [40] (e.g., “I am content with the success I have achieved in my career”). We chose this scale because it could capture essential aspects of career satisfaction effectively and proved to be trustworthy in various settings [41]. The scale was proven to be highly reliable and valid in different studies, especially when the instruments were used with Chinese samples [2]. Cronbach’s coefficient was 0.92 for the scale in this study. The detailed items of the questionnaire can be found in Appendix A.

#### 2.2.2. Professional Identity

A teachers’ professional identity scale including primary, middle, and high school teachers was used in this study [42]. The scale includes 18 items on four dimensions: professional values (e.g., “I think the teaching profession is very important for the promotion of individual human development.”), role values (e.g., “I am proud of being a teacher.”), professional belonging (e.g., “I care what others think of teacher community.”) and professional behavior tendency (e.g., “I can complete the teaching work carefully.”). The items were also scored using a 5-point Likert scale (1 meaning very strongly disagree, 5 meaning very strongly agree). The higher the score, the higher the degree of identification with the engaged profession. This scale was also proven to be highly reliable and valid in different studies, especially when the instruments were used with Chinese samples [16,19]. Cronbach’s coefficient was 0.93 for this scale in this study. The detailed items of the questionnaire can be found in Appendix B.

#### 2.2.3. Psychological Empowerment

Teachers’ psychological empowerment was assessed by a 5-point Likert scoring system, named the Psychological Empowerment Scale (PES), used by [14]. The scale includes 12 items on the following four dimensions: meaning (e.g., “The work I do is very important to me.”), competence (“I am confident about my ability to do my job.”), self-determination (“I have significant autonomy in determining how I do my job.”) and impact (e.g., “My impact on what happens in my department is large.”). Again, this scale was proven to be highly reliable and valid in different studies, especially when the instruments were used with Chinese samples [43,44]. Cronbach’s coefficient was 0.90 for this scale in this study. The detailed items of the questionnaire can be found in Appendix C.

#### 2.2.4. Work Engagement

Teacher work engagement was based on the one-factor (9-item) Utrecht Work Engagement Scale (UWES), which was developed by Schaufeli and Bakker [45]. The scale is scored by 7 Likert points (1 meaning never, 7 meaning always). A higher score in the UWES means a higher work engagement. The UWES was also proven to be highly reliable and valid in different studies, especially when the instruments were used with Chinese samples [46]. The Cronbach’s coefficient was 0.93 for the UWES in the current study. The detailed items of the questionnaire can be found in Appendix D.

### 2.3. Procedure

We recruited subjects on a reliable Chinese data-collection platform which is similar to Qualtrics Online Sample (www.credamo.com) (accessed on 1 June 2021). First, we created and saved the questionnaires required for the study to this platform. Second, before publishing the questionnaires, we simulated answers; after amending the questionnaires, we published them to the platform. Finally, the participants completed the questionnaires via the online survey platform. Prior to their responses, we ensured that their answers were anonymous and confidential, and we confirmed that the data collected would only be used for this specific academic research. After participants read the informed consent form and survey guidelines, they took approximately 20 min to complete the four questionnaires. The study was authorized by the Academic Ethics Committee of Zhejiang Normal University (Protocol code: 20210069, approved 1 April 2021), and it was conducted according to the Declaration of Helsinki.

### 2.4. Statistical Analysis Strategy

In the present study, we used SPSS 23.0 and the PROCESS macro v3.3 [47] for data analysis and processing. The preliminary analysis included descriptive statistics and correlation analysis between the main variables, reflecting the relationship between the main variables. We then ran the SPSS (Model 4) PROCESS macro to examine the mediating effects of psychological empowerment and work engagement between teacher professional identity and career satisfaction. Finally, the SPSS (Model 6) PROCESS macro was used to test the serial mediation effects of psychological empowerment and work engagement. We performed the bootstrapping method to calculate confidence intervals. The test was based on 5000 bootstrap samples to calculate whether the mediating effects were significant at 95%.

## 3. Result

### 3.1. Description and Correlation Statistics

We observed some demographic variables (e.g., gender, age, teaching age) positively correlated with main variables. Hence, like previous studies [31,35], we entered these demographic variables as covariates in subsequent analyses. Table 1 presents the descriptive statistics and the correlations among the main variables. We found that professional identity was positively associated with psychological empowerment, work engagement, and career satisfaction. Psychological empowerment was also positively associated with work engagement, and career satisfaction. In addition, the analyses present a positive correlation between work engagement and career satisfaction.

### 3.2. The Mediating Role of Psychological Empowerment

The present study ran the SPSS (Model 4) PROCESS macro to examine the mediating effect of psychological empowerment (Hypothesis 2) between professional identity and career satisfaction. The analyses revealed that professional identity is positively related to career satisfaction (*β* = 0.22, *p* < 0.001) and psychological empowerment (*β* = 0.58, *p* < 0.001). Meanwhile, psychological empowerment is also positively related to career satisfaction (*β* = 0.55, *p* < 0.001). These analyses demonstrate that psychological empowerment plays a partial mediating role between professional identity and career satisfaction (indirect effect = 0.55, *SE* = 0.03, 95% CI = [0.50, 0.61]). Generally, this model accounted for 58.95% of the total effect, which supports Hypothesis 2. Details of the analyses are shown in Table 2.

**Hypothesis** **1.** *Teacher professional identity is associated with their career satisfaction*.

**Hypothesis** **2.** *The relationship between professional identity and career satisfaction may be mediated by psychological empowerment*.

**Hypothesis** **3.** *Work engagement plays a mediating role between professional identity and career satisfaction*.

**Hypothesis** **4.** *Psychological empowerment and work engagement have an impact on the role of professional identity on teacher career satisfaction in a serial mediation model*.

### 3.3. The Mediating Role of Work Engagement

The present study ran the SPSS (Model 4) PROCESS macro to examine the mediating effect of work engagement (Hypothesis 3) between professional identity and career satisfaction. The analyses revealed that professional identity is positively associated with career satisfaction (*β* = 0.21, *p* < 0.001) and work engagement (*β* = 0.62, *p* < 0.001), and work engagement is positively associated with career satisfaction (*β* = 0.52, *p* < 0.001). These analyses demonstrate that work engagement plays a partial mediating role between professional identity and career satisfaction (indirect effect = 0.57, *SE* = 0.04, 95% CI = [0.50, 0.64]). Generally, this model accounted for 60.32% of the total effect, which supports Hypothesis 3. Details of the analyses are summarized in Table 3.

### 3.4. Testing the Multiple Mediation Model

**Figure 2:**
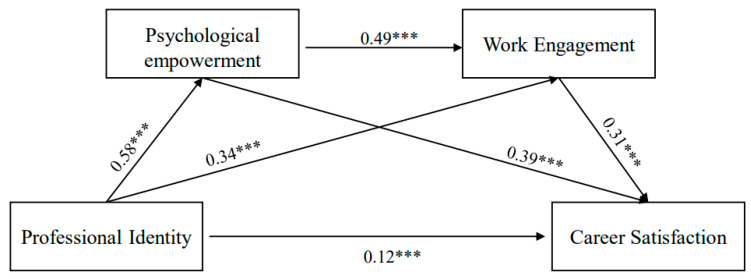
The pathway coefficients of multiple mediation model. *** *p* < 0.0014.

The present study ran the SPSS (Model 4) PROCESS macro to examine the multiple mediation model among the main variables (Hypothesis 4). The results show that the pathways for “professional identity → psychological empowerment → career satisfaction” (indirect effect = 0.40, 95% CI = [0.34, 0.45]), and “professional identity → work engagement → career satisfaction” (indirect effect = 0.18, 95% CI = [0.14, 0.22]) are significant, which indicates that psychological empowerment and work engagement mediate the relationship between professional identity and career satisfaction. Similarly, the sequential pathway for “professional identity → psychological empowerment → work engagement → career satisfaction” (indirect effect = 0.15, 95% CI = [0.12, 0.18]) suggests that a higher level of professional identity is serially related to higher levels of psychological empowerment (*β* = 0.58, *p* < 0.001), work engagement (*β* = 0.49, *p* < 0.001), and career satisfaction (*β* = 0.31, *p* < 0.001). In addition, the direct pathway for “professional identity → career satisfaction” is also significant (*β* = 0.12, *p* < 0.001). Therefore, psychological empowerment and work engagement play mediating roles between professional identity and career satisfaction, both in parallel and sequentially. Generally speaking, the multiple mediation model accounted for a significant amount of variance in primary and secondary school teacher career satisfaction, which supports Hypothesis 4. Further details about the analyses are summarized in Table 4 and Figure 2.

## 4. Discussion

The present study aims to gain a deeper understanding of the underlying mechanism between professional identity and career satisfaction. Based on theories and empirical studies outlined above, the present study proposed that psychological empowerment, followed by work engagement, would serially mediate the relationship between professional identity and teacher career satisfaction. Notwithstanding that much previous research explored how professional identity plays a role in career satisfaction, such studies usually only examined the relationship between professional identity and one or two variables. For example, Helen et al. explored Australian occupational therapists’ professional identity, the factors affecting career satisfaction and job burnout, and their relationship [48]. In addition, few empirical studies were conducted by previous researchers to investigate the mediating role between professional identity and career satisfaction. In the present study, these analyses confirmed the hypothesized multiple mediator models (psychological empowerment and work engagement). These findings emphasize the essential role of psychological empowerment and work engagement in the impact path of professional identity and career satisfaction. Specifically, the higher the level of professional identity of teachers, the higher their psychological empowerment will be, which leads to increased work engagement and, ultimately, improved career satisfaction.

### 4.1. Relationship between Professional Identity and Career Satisfaction

We confirmed that professional identity is significantly and positively related to teacher career satisfaction, supporting Hypothesis 1. This conclusion is consistent with those of previous studies. For example, Ye and Zheng found that the influence of professional identity on the career satisfaction of rural primary school principals is moderated by emotional intelligence [12]. In addition, our results validate the conservation of resources (COR) theory [49], indicating that professional identity could reduce work pressure and increase resources to cope with adverse working conditions, which improves career satisfaction [50,51,52]. According to COR theory, science teacher professional identity can provide them with a valuable psychological resource to achieve self-development and relieve stress [53]. When teachers identify with their profession, they are more likely to view work as fun and challenging than pressure and demand. Based on COR theory, some researchers believe that individuals with high levels of professional identity are able to find their value in the organization and will strive to seek and retain what they value [30,49], thus ultimately improving their career satisfaction [54]. Therefore, when teachers have a positive professional identity, educating students will be regarded as significant. Teachers gain a positive sense of accomplishment from it [54], which will provide them with positive and effective psychological resources to work better.

### 4.2. Psychological Empowerment as a Mediator

The present study confirms that the relationship between professional identity and career satisfaction is mediated by psychological empowerment, supporting Hypothesis 2. Teacher psychological empowerment refers to teachers being confident that they can do their jobs well and believing that their work is meaningful and valuable [14,55]. Teacher professional identity is considered a feeling of recognition held by teachers for the profession of “teacher” [56]. Therefore, when teachers have a higher level of recognition of their profession, they will think more of their work is meaningful and valuable. That is, they will have a higher level of psychological empowerment. Additionally, we can also explain the mediating effect of psychological empowerment on professional identity and career satisfaction from the perspective of COR theory [49]. Psychological empowerment, as a synthesis of psychological states or cognitions experienced by an individual, can be used as an intrinsic motivation for oneself [57], increasing the individual’s psychological resources. When teachers have a high level of psychological empowerment, they will see their work as meaningful and worth their effort, thereby increasing their psychological resources and improving their career satisfaction.

### 4.3. Work Engagement as a Mediator

The present research analyses confirm that teachers with a high level of professional identity tend to work hard, which in turn leads to a higher level of career satisfaction, suggesting that work engagement mediates the relationship between professional identity and career satisfaction, supporting Hypothesis 3. Present results show that work engagement is positively related to teachers career satisfaction [58]. This conclusion is consistent with previous studies on the mediating effect of work engagement on professional identity (especially the dedication component of work engagement) and career satisfaction [31,32]. Moreover, our findings validate the Job Demands Resource Model (JD–R model) [45,59]. Through the motivational stimulation process, when individuals feel the meaning of work, they will continue to demonstrate work vitality, dedication, and focus, thereby generating work engagement [60] and improving career happiness and satisfaction [61,62]. Therefore, teachers can fully devote themselves to their work when they have a professional identity in terms of the profession of “teacher”, which will improve their professional identity.

### 4.4. Psychological Empowerment and Work Engagement as Mediators

The present research investigated the way in which professional identity affects teacher career satisfaction through psychological empowerment and career satisfaction. Therefore, Hypothesis 4 of the present study is finally confirmed. Self-Determination Theory showed that the availability of psychological resources to an individual is an important factor affecting their behavior [63,64]. Meanwhile, they believed that psychological empowerment reflects personal needs that require satisfying, which may mean that individuals with high levels of psychological empowerment could generate more psychological resources. According to COR theory [49], improving an individual’s professional identity effectively supplements psychological resources [65,66]. The increase in psychological resources will increase the individual’s work enthusiasm and thus improve work engagement and career satisfaction [30,67]. The present research supports and confirms the Self-Determination and COR theories by investigating the mediating mechanism of professional identity and teacher career satisfaction. Teachers with a higher level of professional identity will generate many more valuable psychological resources and gain higher levels of psychological empowerment, becoming more active at work. Obtaining such resources will ultimately improve career satisfaction.

### 4.5. Implications for Practice

In addition to its contributions to theory, the conclusion of the present research also suggests several practical implications. Psychological empowerment is a vital factor that explains how professional identity affects teacher career satisfaction. Teachers with high levels of psychological empowerment can take the initiative to improve their abilities, increase psychological resources and are more able to relieve the pressure brought by work. Therefore, schools should hold regular training to provide teachers with practical methods to improve their psychological empowerment. In addition, work engagement is another vital factor that explains how professional identity affects teacher career satisfaction. Improving levels of teacher work engagement may increase the levels of their career satisfaction. Hence, schools should ask teachers to set staged work goals and give positive feedback, such as rewards, when they achieves these. On the whole, the present study provides some possible entry points for improving teacher career satisfaction.

### 4.6. Limitations and Future Research

Despite its significant contributions, this research has several limitations. First, the research was a cross-sectional survey design; although many previous studies [68,69,70] conducted mediation analysis of cross-sectional data points, cross-sectional studies can only explain the correlations between variables and cannot make inferences about causality. Future research should adopt interaction analysis to analyze the data, which would be more suitable for cross-sectional data. Alternatives would be to use a longitudinal design to investigate the casual relationship between the main variables or to conduct an experiment to examine casual relationships. Second, all the data were collected by the self-reported method, which may cause recall bias and be influenced by social expectations. Hence, future research could develop and adopt more objective professional identity and career satisfaction measures. Third, career satisfaction can be affected by two main and significant aspects: an individual’s own and an organization’s aspects. In this study, professional identity, psychological empowerment, and work engagement are factors pertaining to the individual aspect. Future research could, therefore, examine the impact of organizational factors on career satisfaction. Fourth, even though our sample was relatively large, the proportion of females in the sample was high. Additionally, almost all samples come from the same region, so other researchers should repeat the study with larger samples in different research regions in the future. Finally, it was valuable to discuss the effect of national (or regional) culture on career satisfaction. However, in the current study, we only collected data from Zhejiang, China. Hence, it was difficult for us to discuss the effect of national (or regional) culture on career satisfaction. Future studies could involve comparative studies between different countries or a meta-analysis to explore the effect of national (or regional) culture on career satisfaction.

## 5. Conclusions

The present study indicated that there is a positively associated relationship between professional identity and teacher career satisfaction. More importantly, professional identity can indirectly make an impact on teacher career satisfaction through the single mediating effects of psychological empowerment and work engagement, and the chain mediating effect, by improving the level of psychological empowerment, thereby increasing work engagement.

## Figures and Tables

**Table 1:** Means, Standard Deviations, and Inter-Correlations between the Main Variables (N = 2014).

	*M*	*SD*	1	2	3	4
1. PI	4.5	0.49	1	*—*	*—*	*—*
2. PE	3.89	0.68	0.59 **	1	*—*	*—*
3. WE	3.90	0.78	0.63 **	0.69 **	1	*—*
4. CS	3.83	0.85	0.54 **	0.67 **	0.65 **	1

Note: ** *p* < 0.01. Abbreviations: PI, professional identity; PE, psychological empowerment; WE, work engagement; CS, career satisfaction.

**Table 2:** Testing Psychological Empowerment as a Mediator in the Relationship between Professional Identity and Career Satisfaction (N = 2104).

Criterion	Predictors	*R*	*R*²	*F*	*β*	Boot LLCI	Boot ULCI	*t*
PE	PI	0.59	0.35	284.70 ***	0.58	0.76	0.88	33.13 ***
CS	PI	0.70	0.49	400.32 ***	0.22	0.32	0.46	11.47 ***
	PE				0.55	0.62	0.72	28.10 ***

Note: *** *p* < 0.001. Standardized regression coefficients are reported. Bootstrap sample size = 5000. Abbreviations: PI, professional identity; PE, psychological empowerment; CS, career satisfaction LL, low limit; UL, upper limit; CI, confidence interval.

**Table 3:** Testing Work engagement as a Mediator in the Relationship between Professional Identity and Career Satisfaction (N = 2104).

Criterion	Predictors	*R*	*R*²	*F*	*β*	Boot LLCI	Boot ULCI	*t*
WE	PI	0.64	0.41	368.58 ***	0.62	0.92	1.07	37.25 ***
CS	PI	0.67	0.46	350.72 ***	0.21	0.29	0.46	10.28 ***
	WE				0.52	0.51	0.62	24.79 ***

Note: *** *p* < 0.001. Standardized regression coefficients are reported. Bootstrap sample size = 5000. Abbreviations: PI, professional identity; WE, work engagement; CS, career satisfaction; LL, low limit; UL, upper limit; CI, confidence interval.

**Table 4:** Testing the Pathways of the Multiple Mediation Model.

Effect	*β*	*SE*	95%Boot LLCI	95%Boot ULCI
Direct effects				
PI→PE	0.58 ***	0.02	0.76	0.88
PI→WE	0.34 ***	0.03	0.47	0.62
PE→WE	0.49 ***	0.02	0.51	0.60
PI→CS	0.12 ***	0.03	0.13	0.27
PE→CS	0.39 ***	0.03	0.43	0.54
WE→CS	0.31 ***	0.02	0.28	0.40
Indirect effects				
PI→PE→CS	0.40	0.03	0.34	0.45
PI→WE→CS	0.18	0.02	0.14	0.23
PI→PE→WE→CS	0.15	0.02	0.12	0.18

Note: *** *p* < 0.001. Standardized regression coefficients are reported. Bootstrap sample size = 5000. Abbreviations: PI, professional identity; PE, psychological empowerment; WE, work engagement; CS, career satisfaction; LL, low limit; UL, upper limit; CI, confidence interval.

## Data Availability

The authors have specified no data sets for the following reason: The data that has been used is confidential. Due to the sensitive nature of the questions asked in this study, survey respondents were assured raw data would remain confidential and would not be shared.

## Appendix A. Career Satisfaction Items

**Q**	**Items**	**1**	**2**	**3**	**4**	**5**
1.	I am satisfied with the success I have achieved in my career					
2.	I am satisfied with the progress I have made toward meeting my overall career goals					
3.	I am satisfied with the progress I have made toward meeting my goals for income					
4.	I am satisfied with the progress I have made toward meeting my goals for advancement					
5.	I am satisfied with the progress I have made toward meeting my goals for the development of new skills					

## Appendix B. Professional Identity Items

**Q**	**Items**	**1**	**2**	**3**	**4**	**5**
1.	I think the teaching profession is very important to promote the development of human beings					
2.	I care about how others perceive the teaching profession					
3.	As a teacher, I often feel respected					
4.	I can complete my teaching work seriously					
5.	I am suitable for teaching					
6.	Being a teacher can realize the value of my life					
7.	I am proud of being a teacher					
8.	I will be gratified when I see or hear words praising the teaching profession					
9.	I am able to complete work tasks on time					
10	When introducing myself, I like to mention that I am a teacher					
11.	I think the teaching profession is one of the most important professions in the social division of labor					
12.	I care what others think of the teacher community					
13	I am proactive in creating harmonious co-worker relationships					
14.	I think the teaching profession is very important to promote the individual development of human beings					
15.	I can take my work within my area of responsibility seriously					
16.	In order to maintain the normal teaching order of the school, I will abide by those informal systems					
17.	I feel insulted when someone baselessly accuses teacher groups					
18.	I think the work of teachers is important in promoting the growth and development of students					

## Appendix C. Psychological Empowerment Items

**Q**	**Items**	**1**	**2**	**3**	**4**	**5**
**Meaning**						
1.	The work I do is very important to me.					
2.	My job activities are personally meaningful to me					
3.	The work I do is meaningful to me					
**Competence**						
4.	I am confident about my ability to do my job					
5.	I am self-assured about my capabilities to perform my work activities					
6.	I have mastered the skills necessary for my job					
**Self-Determination**						
7.	I have significant autonomy in determining how I do my job					
8.	I can decide on my own how to go about doing my work					
9.	I have considerable opportunity for independence and freedom in how I do my job					
**Impact**						
10.	My impact on what happens in my department is large					
11.	I have a great deal of control over what happens in my department					
12.	I have significant influence over what happens in my department					

## Appendix D. Work Engagement Items

**Q**	**Items**	**1**	**2**	**3**	**4**	**5**	**6**	**7**
**Vigor**								
1.	I feel myself bursting with energy in my work							
2.	I feel strong and energized when I work							
3.	I’m passionate about my work							
4.	Work inspires me							
5.	As soon as I wake up in the morning, I want to go to work							
6.	When work is stressful, I feel happy							
7.	I am proud of the work I do							
8.	I am immersed in my work							
9.	I will reach a state of forgetfulness when working							
